# Serological Response to Influenza Vaccination among Children Vaccinated for Multiple Influenza Seasons

**DOI:** 10.1371/journal.pone.0051498

**Published:** 2012-12-11

**Authors:** Sajjad Rafiq, Margaret L. Russell, Richard Webby, Kevin Fonseca, Marek Smieja, Pardeep Singh, Mark Loeb

**Affiliations:** 1 Department of Pathology, McMaster University, Hamilton, Ontario, Canada; 2 Department of Community Health Sciences, University of Calgary, Calgary, Alberta, Canada; 3 Department of Infectious Diseases, St. Jude Children’s Research Hospital, Memphis, Tennessee, United States of America; 4 Provincial Laboratory for Public Health, Calgary, Alberta, Canada; 5 Departments of Pathology and Molecular Medicine and Clinical Epidemiology and Biostatistics, McMaster University, Hamilton, Ontario, Canada; University of Hong Kong, Hong Kong

## Abstract

**Background:**

To evaluate if, among children aged 3 to 15 years, influenza vaccination for multiple seasons affects the proportion sero-protected.

**Methodology/Principal Findings:**

Participants were 131 healthy children aged 3–15 years. Participants were vaccinated with trivalent inactivated seasonal influenza vaccine (TIV) over the 2005–06, 2006–07 and 2007–8 seasons. Number of seasons vaccinated were categorized as one (2007–08); two (2007–08 and 2006–07 or 2007–08 and 2005–06) or three (2005–06, 2006–07, and 2007–08). Pre- and post-vaccination sera were collected four weeks apart. Antibody titres were determined by hemagglutination inhibition (HAI) assay using antigens to A/Solomon Islands/03/06 (H1N1), A/Wisconsin/67/05 (H3N2) and B/Malaysia/2506/04. The proportions sero-protected were compared by number of seasons vaccinated using cut-points for seroprotection of 1∶40 vs. 1∶320. The proportions of children sero-protected against H1N1 and H3N2 was high (>85%) regardless of number of seasons vaccinated and regardless of cut-point for seroprotection. For B Malaysia there was no change in proportions sero-protected by number of seasons vaccinated; however the proportions protected were lower than for H1N1 and H3N2, and there was a lower proportion sero-protected when the higher, compared to lower, cut-point was used for sero-protection.

**Conclusion/Significance:**

The proportion of children sero-protected is not affected by number of seasons vaccinated.

## Introduction

Annual influenza vaccination for all children aged 6 to 59 months was recommended in 2004 for Canada, [1] and in 2006 for the United States [2]. In the United States, this recommendation was expanded to include all children aged 5 to 18 years beginning with the 2008–09 season [3]. One concern raised about vaccinating children annually against influenza is a ceiling effect of the immune response to the vaccine components; namely, with repeated annual vaccination with a different influenza antigen(s), the antibody response will plateau over time [4], [5]. This concern is based on the concept of original antigenic sin, where memory B cells from the primary infection interfere with the naive B cell response to altered epitopes [6]–[8]. As sero-protection is the key parameter of public health importance [9], there is a need for further data to examine this possibility.

In order to better define the effect of repeated vaccination with inactivated influenza vaccine on sero-protection in children, we conducted a prospective study. Our goal was to evaluate the effect of repeated vaccination (i.e., vaccination for more than one influenza season) with recommended vaccine antigens and to measure the effect on sero-protection against these antigens in children.

## Methods

### Ethics Statement and Role of the Funding Source

The study was approved by the Conjoint Health Research Ethics Board of the University of Calgary (Ethics ID 18970) and McMaster University HHS/FHS Research Ethics Board (REB project # 07-376). Informed written consent was obtained from the parents/guardians of the children. Written informed assent was additionally obtained from all children aged 7 years or older. The funding source had no role in study design; collection, analysis or interpretation of data; writing of the report; nor decision to submit the paper for publication.

### Participants and Intervention

Participants were healthy children (i.e., no underlying chronic medical conditions) aged 3–15 years residing on central Alberta Hutterite colonies who received study vaccine in 2007–08. Each year from the 2005/06 to 2007/08 influenza seasons, participating children were vaccinated (as part of a pilot study) with the trivalent inactivated seasonal influenza vaccine (TIV) of the year, according to the age-specific recommendations of the Canadian National Advisory Committee on Immunization [10]–[12]. Children aged less than 9 years who had not been previously vaccinated were given two age-appropriate doses of vaccine four weeks apart. All participants were offered influenza vaccination each year. In each year some new participants entered study as they attained the age for study eligibility, all were offered the influenza vaccine of the year. Vaccines were administered intramuscularly (deltoid) using 5/8 inch or 1 inch needles. Children were excluded from vaccination if there was a history of anaphylactic reaction to a previous dose of influenza vaccine; known IgE-mediated hypersensitivity to eggs manifested as hives, swelling of the mouth and throat, difficulty in breathing, hypotension, or shock; or Guillain-Barré syndrome within eight weeks of a previous influenza vaccine.

Children were classified as having been vaccinated for three seasons if they had been vaccinated by the study team in each of 2005–06, 2006–07, and 2007–08. Those vaccinated for two seasons were vaccinated in 2007–08 and 2006–07 or in 2007–08 and 2005–06. Those vaccinated for one season were vaccinated only in 2007–08. Table 1 displays the vaccines used for each year (none contain any adjuvant).

**Table 1:** Vaccine antigens by year.

Year	Vaccines used	Vaccine antigens
2005–06	Fluviral® GlaxoSmithKline Inc Lot 3FV27511	A/New Caledonia/20/99 (H1N1)
		A/New York/55/04 (H3N2)
		B/Jiangsu/10/03^a^
2006–07	Fluviral® GlaxoSmithKline Inc Lots C2753AA and C2755AA	A/New Caledonia/20/99
		A/Wisconsin/67/05^b^
		B/Malaysia/2506/04^c^
2007–08	Vaxigrip® Sanofi Pasteur Lot C2984AA	A/Solomon Islands/3/06 (H1N1)-like strain (A/Solomon Islands/3/06 IVR-145)
		A/Wisconsin/67/05 (H3N2)-like strain (A/Wisconsin/67/05 NYMC X-161B)
		B/Malaysia/2506/04-like strain (B/Malaysia/2506/04)

a A/New York/55/04 is antigenically equivalent to the A/California/7/04 (H3N2) virus strain; B/Jiangsu/10/03 is antigenically equivalent to the B/Shanghai/361/02 virus strain [10] and is of the B Yamagata lineage [1].

b A/Hiroshima/52/05 is antigenically equivalent to the A/Wisconsin/67/05 virus strain [11].

c B/Malaysia/2506/04 belongs to the B/Victoria/02/87 lineage [11].

Pre- and post-vaccination blood samples from participants were collected four weeks apart, centrifuged and the serum stored at −20°C before parallel testing against the influenza A and B vaccine components. Antibody titres to each influenza type and subtype were determined by the hemagglutination inhibition assay using antigens to A/Solomon Islands/03/06 (H1N1), A/Wisconsin/67/05 (H3N2) and B/Malaysia/2506/04. All subject and control sera were pre-treated with receptor-destroying enzyme prior to titration. Doubling dilutions from 1∶20 to 1∶2560 were performed for all subject and serum control samples. Acute and convalescent sera were tested in parallel for comparability of titres. The assay was performed using turkey red blood cells (Rockland Immunochemicals, Philadelphia, Pennsylvania) as the indicator together with 4HA units of the respective influenza antigen as described in the WHO method [13]. The endpoint titre was the reciprocal of the highest dilution to show complete inhibition of hemagglutination. Appropriate controls were run with each batch of samples to identify non-specific agglutination. Sero-protection status was defined by an antibody measurement reciprocal titre of greater than or equal to 40, found either pre- or post-vaccination [14]; however, as this may not be an appropriate cut-point for children, we also explored the impact of defining sero-protection using a value of 320 which may be a more appropriate cut point for sero-protection [15] among children.

### Statistical Analyses

For each age-group by antigen, we compared the proportions of children that sero-converted by season first using a cut-point of 40 and then of 320. For all analyses we used an unadjusted alpha of 0.05.

Statistical analyses were done using STATA v10 (Statacorp, College Station, TX, U.S.A. and SAS v9 (SAS Institute, Cary NC).

## Results

### Description of Study Population

Although consent was received for vaccination of 138 children in 2007/08, pre-post vaccination sera were available only for 131. Table 2 displays the age distribution and number of seasons vaccinated for the 131 children for whom serological data were available. The mean age was 8.9 years; the majority of children were aged 6–15 years (80.9%). There were 64 females (48.9%) and 67 males. The largest proportion of children had been vaccinated for all three seasons (58.0%); 28.3% had been vaccinated for only one season and 13.7% for two seasons (Table 2).

**Table 2:** Numbers of children (2007–08 season) with pre-post vaccination sera by age-group and number of seasons vaccinated.

	Age group*		Totals
Number of seasons vaccinated	3–5 years (%)	6–15 years (%)	
Vaccinated only one season (2007–08)	11 (44)	26 (24.5)	37
Vaccinated two seasons OR (2007–08 and 2006–072007–08 and 2005–06)	9 (36)	9 (8.5)	18
Vaccinated three seasons (2007–08 and 2006–07and 2005–06)	5 (20)	71 (67.0)	76
Total	25	106	131
P-Value (Fisher’s exact test)	<<0.001		

* Age-group as of November 1, 2007.

### Sero-Protection

As can be seen in Table 3, using a cut-point of 40 for sero-protection, similar proportions of children were sero-protected for both age groups for both the H1N1 and H3N2 antigens regardless of number of seasons vaccinated. Similarly, although a smaller proportion of those aged 6–15 years than those aged 3–5 years were sero- protected against B/Malaysia/2506/04, there was no significant difference in proportions sero-protected by number of seasons vaccinated. Table 4 displays the results for a cut-point of 320. The proportions of children sero-protected against B/Malaysia/2506/04 were less than the proportions sero-protected against either H1N1 or H3N2 for both children aged 3–5 years and those aged 6–15 years. Indeed for the higher cut-point, the large majority of children, especially those aged 6–15 years are no longer classified as sero-protected against B/Malaysia/2506/04. However, regardless of age-group, there was no significant difference in proportions sero-protected by number of seasons vaccinated for all three antigens even with a 320 cut-point.

**Table 3:** Age−specific proportions of children sero-protected (post-vaccination titre cut point > = 40) by N seasons vaccinated.

Strain	Age group*	Vaccinated onlyone season(2007–08)	Vaccinated two seasons (2007–08and 2006–07 OR2007–08 and2005–06)	Vaccinated three seasons (2007–08 and 2006–07 and 2005–06)	P-Value
A/Solomon Islands/3/2006 (H1N1)	3–5 years				
	n(%)	11 (100.0)	9 (100.0)	5 (100.0)	1.00
	6–15 years				
	n (%)	25 (96.2)	9 (100.0)	71 (100.0)	0.21
A/Wisconsin/67/05 (H3N2)	3–5 years				
	n (%)	11 (100.0)	9 (100.0)	5 (100.0)	1.00
	6–15 years				
	n (%)	26 (100.0)	9 (100.0)	71 (100.0)	1.00
B/Malaysia/2506/04	3–5 years				
	n (%)	11 (100.0)	9 (100.0)	4 (80.0)	0.13
	6–15 years				
	n (%)	19 (73.1)	7 (77.8)	56 (78.9)	0.83

* Age-group as of November 1, 2007.

**Table 4:** Age−specific proportions of children sero-protected (post-vaccination titre cut point > = 320) by N seasons vaccinated.

Strain	Age group*	Vaccinated only one season (2007–08)	Vaccinated two seasons (2007–08 and 2006–07 OR 2007–08 and 2005–06)	Vaccinated three seasons (2007–08 and 2006–07 and 2005–06)	P-Value
A/Solomon Islands/3/2006 (H1N1)	3–5 years				
	n (%)	11 (100.0)	9 (100.0)	5 (100.0)	1.00
	6–15 years				
	n (%)	23 (88.5)	9 (100.0)	70 (98.6)	0.06
A/Wisconsin/67/05 (H3N2)	3–5 years				
	n (%)	10 (90.9)	8 (88.9)	5 (100.0)	0.75
	6–15 years				
	n (%)	24 (92.3)	8 (88.9)	69 (97.2)	0.39
B/Malaysia/2506/04	3–5 years				
	n (%)	6 (54.5)	3 (33.3)	4 (80.0)	0.24
	6–15 years				
	n (%)	8 (30.8)	2 (22.2)	13 (18.3)	0.41

* Age-group as of November 1, 2007.

## Discussion

We found that most children were sero-protected against influenza regardless of number of seasons vaccinated; however, this proportion was less for influenza B than for influenza A. The impact of using a cut-point of 320 rather than the value of 40 that has been used in adult studies is important to understanding the serological response of children to vaccination; not surprisingly the proportion of children protected declines when the higher cut-point is used, particularly for influenza B. As sero-protection is the most relevant indicator from a public health perspective, future studies should use this higher cut-point in their analyses.

The strengths of this study include a larger sample and wider age-span than that of Zeman [5], who had observations for only 21 children aged 3–9 years; however despite this we still had small numbers in some age strata. An additional strength was our exploration of higher cut-points for sero-protection: our findings of a lack of impact on sero-protection of number of seasons vaccinated was robust to cut-point and to age-group.

There are limitations to our study: small sample sizes in some strata result in limited power, and we could not account for the effect of prior natural infection compared to immunization. Nevertheless, our data demonstrate that lack of priming did not have a detrimental effect on seroprotection, which is most relevant from a public health perspective. Finally, the changing status of the antigens in both vaccines and circulating strains makes interpretation of study findings challenging.

### Conclusion

The proportion of children who are sero-protected against influenza B was less than for either influenza A (H1N1) or A (H3N2).

## References

[1] National Advisory Committee on Immunization (2004) Statement on Influenza Vaccination for the 2004–2005 Season. Canada Communicable Disease Report 30: 1–32. 15239483

[2] Advisory Committee on Immunization Practices (2006) Prevention and control of influenza: recommendations of the Advisory Committee on Immunization Practices (ACIP). MMWR Recommendations & Reports 55: 1–42. 16874296

[3] Advisory Committee on Immunization Practices (2008) Prevention and Control of Influenza 2008. MMWR: Morbidity & Mortality Weekly Report 57: 1–60. 18685555

[4] Gross PA, Sperber SJ, Donabedian A, Dran S, Morchel G, et al (1999) Paradoxical response to a novel influenza virus vaccine strain: the effect of prior immunization. Vaccine 17: 2284–2289 doi:DOI: 10.1016/S0264-410X(98)00478-2 10403596 10.1016/s0264-410x(98)00478-2

[5] Zeman AM, Holmes TH, Stamatis S, Tu W, He XS, et al (2007) Humoral and cellular immune responses in children given annual immunization with trivalent inactivated influenza vaccine. Pediatric Infectious Disease Journal 26: 107–115. 17259871 10.1097/01.inf.0000253251.03785.9b

[6] Morens DM, Burke DS, Halstead SB (2010) The wages of original antigenic sin. Emerging Infectious Diseases 16: 1023–1024. 20507764 10.3201/eid1606.100453 PMC3086238

[7] Kim JH, Skountzou I, Compans R, Jacob J (2009) Original Antigenic Sin Responses to Influenza Viruses. The Journal of Immunology 183: 3294–3301. 19648276 10.4049/jimmunol.0900398 PMC4460008

[8] Francis T Jr (1960) On the Doctrine of Original Antigenic Sin. Proceedings of the American Philosophical Society 104: 572–578.

[9] Beyer WE, Palache AM, Luchters G, Nauta J, Osterhaus AD (2004) Seroprotection rate, mean fold increase, seroconversion rate: which parameter adequately expresses seroresponse to influenza vaccination? Virus Research 103: 125–132 doi:DOI: 10.1016/j.virusres.2004.02.024 15163500 10.1016/j.virusres.2004.02.024

[10] National Advisory Committee on Immunization (2005) Statement on influenza vaccination for the 2005–2006 season. Canada Communicable Disease Report 31: 1–32. 15959943

[11] National Advisory Committee on Immunization (2006) Statement on influenza vaccination for the 2006–2007 season. Canada Communicable Disease Report 32: 1–28. 16805064

[12] National Advisory Committee on Immunization (2007) Statement on influenza vaccination for the 2007–2008 season. Canada Communicable Disease Report 33: 1–13. 18163241

[13] World Health Organization (2002) WHO Manual on Animal Influenza Diagnosis and Surveillance. http://whqlibdoc.who.int/hq/2002/WHO_CDS_CSR_NCS_2002.5.pdf.

[14] Hannoun C, Megas F, Piercy J (2004) Immunogenicity and protective efficacy of influenza vaccination. Virus Research 103: 133–138 doi:10.1016/j.virusres.2004.02.025 15163501 10.1016/j.virusres.2004.02.025

[15] Black S, Nicolay U, Vesikari T, Knuf M, Del Giudici G, et al (2011) Hemagglutination inhibition antibody titers as a correlate of protection for inactivated influenza vaccines in children. Pediatric Infectious Disease Journal 30: 1081–1085. 21983214 10.1097/INF.0b013e3182367662

